# From Environment to Man: Genome Evolution and Adaptation of Human Opportunistic Bacterial Pathogens

**DOI:** 10.3390/genes3020191

**Published:** 2012-03-26

**Authors:** Fabien Aujoulat, Frédéric Roger, Alice Bourdier, Anne Lotthé, Brigitte Lamy, Hélène Marchandin, Estelle Jumas-Bilak

**Affiliations:** 1 Université Montpellier 1, UMR 5119 (UM2, CNRS, IRD, IFREMER, UM1), équipe Pathogènes et Environnements, Montpellier 34093, France; E-Mails: fabien.aujoulat@univ-montp1.fr (F.A.); frederic.rogerfrederic@laposte.net (F.R.); alice.bourdier@univ-montp1.fr (A.B.); a-lotthe@chu-montpellier.fr (A.L.); b-lamy@chu-montpellier.fr (B.L.); helene.marchandin@univ-montp1.fr (H.M.); 2 Centre Hospitalier Universitaire, Laboratoire d’Hygiène hospitalière, Montpellier 34000, France; 3 Centre Hospitalier Universitaire, Laboratoire de Bactériologie, Montpellier 34000, France

**Keywords:** bacterial genome, opportunistic human pathogens, environment, adaptation, emergence

## Abstract

Environment is recognized as a huge reservoir for bacterial species and a source of human pathogens. Some environmental bacteria have an extraordinary range of activities that include promotion of plant growth or disease, breakdown of pollutants, production of original biomolecules, but also multidrug resistance and human pathogenicity. The versatility of bacterial life-style involves adaptation to various niches. Adaptation to both open environment and human specific niches is a major challenge that involves intermediate organisms allowing pre-adaptation to humans. The aim of this review is to analyze genomic features of environmental bacteria in order to explain their adaptation to human beings. The genera *Pseudomonas*, *Aeromonas* and *Ochrobactrum* provide valuable examples of opportunistic behavior associated to particular genomic structure and evolution. Particularly, we performed original genomic comparisons among aeromonads and between the strictly intracellular pathogens *Brucella* spp. and the mild opportunistic pathogens *Ochrobactrum* spp. We conclude that the adaptation to human could coincide with a speciation in action revealed by modifications in both genomic and population structures. This adaptation-driven speciation could be a major mechanism for the emergence of true pathogens besides the acquisition of specialized virulence factors.

## 1. Introduction

Regarding their pathogenicity towards human beings, bacteria are commonly classified as true (or strict) pathogens and opportunistic (or facultative) pathogens. Opportunistic Bacterial Pathogens (OBPs) can cause infections in patients with underlying conditions such as indwelling devices [1] or diseases such as cystic fibrosis (CF) [2]. More generally, OBPs can cause disease when the host’s resistance is low, whatever the reason and the duration of the host’s failure. This is particularly true for healthcare-associated infections (HAIs), which cause added mortality and healthcare costs in developed countries [3] and increase the burden of resource use in the strained healthcare systems of developing countries [4]. In the last two decades, the impact of HAI and other opportunistic infections has notably increased; many OBPs are now being considered as emerging pathogens. Therefore, understanding the behavior and evolution of opportunistic pathogens remains a major medical challenge. 

One generally considers that antimicrobial resistance and host susceptibility explain the opportunism and pathogenic success of OBPs, as opposed to true pathogens. However, each OBP displays a somewhat specific behavior with a predilection for an underlying pathology or a particular clinical status. The corresponding genetic determinants remain poorly characterized, as do the characteristics of OBP strains, in comparison to specialized environmental strains. OBPs are generally considered as harmless bacteria devoid of specific virulence factors. This is true for several mild OBPs that cause diseases only when patients are deeply immunocompromised, debilitated or subject to invasive procedures. However, notorious OBPs such as *Pseudomonas aeruginosa* are also well known over-armed pathogens that can exhibit a battery of virulence factors [5]. On closer analysis, these so-called virulence factors primarily serve general adaptation purposes, and it is their association that enables OBPs to infect susceptible hosts. In contrast to what is observed in strict pathogens, knockout mutations on corresponding virulence genes do not turn the bacteria avirulent. Some attenuated phenotypes related to virulence can be observed but the opportunistic pathogen behavior of the strain probably remains. 

More generally, all bacteria display different virulent behaviors depending on their hosts, and the distinction between behavior patterns is not clear. Instead, one can observe a continuum from bacterial adaptation to a host to non-specific virulence to specialization as strict pathogens. In this wide array of biodiversity—adaptation and virulence properties—a comprehensive understanding of OBPs is difficult to achieve. Therefore, predicting the impact of emerging pathogens on human health is an almost impossible task. An additional difficulty is the ability of OBPs to change unpredictably in relation to abiotic and biotic factors. Biotic factors involve interrelationships with a myriad of hosts of variable immune status, in dynamic natural or anthropogenic environments, the latter including antimicrobial agents. The pragmatic approach for predicting and preventing pathogen emergence is based on epidemiological surveillance. In the future, the abundance of microbial genomic data, with an emphasis on comparative genomics, should give us a better understanding of evolution, micro-evolution and adaptation in OBPs. Hopefully this will identify traits common to all OBPs, allowing for better surveillance, prevention and treatment of the infections they cause.

This review describes the genomic evolution of OBPs of environmental origin, based on published studies as well as on original genome comparisons. Genomic features will be considered in the context of OBPs' adaptation to human beings. Examples will be taken in order to depict different opportunistic lifestyles in relation to different modes of genomic evolution and adaptation. A synthetic but non-exhaustive table indicating natural habitat, life-styles and pathogenicity for human beings is proposed for most OBPs described in this review in Table 1. 

## 2. Environment is a ‘Nursery’ for Emerging OBPs

Mutualist bacteria, also named commensal bacteria, members of the human microbiota, are good candidates for becoming OBPs because they are already adapted to grow in human beings, and to escape from and/or to be tolerated by the immune system. To a lesser extent, the situation is similar for wild or domestic mammals—associated bacteria causing anthropozoonoses [6,7,8]. Mutualist OBPs can escape from their original niche and cause a variety of infectious complications by reaching sterile or atypical anatomic sites [9]. They can also invade the niches, surpassing in number other members of the community, hence inducing dysbiosis and local infections. Pathogenicity of mutualist strains is related to their strategies to effectively colonize their niche: strategies that can later be opportunistically used for the invasion of other anatomic niches. In the case of anthropozoonoses, large scale transmission between mammals or birds and humans can select new or emerging virulence traits that may include increased invasiveness, enhanced spread, toxin production or antimicrobial drug resistance [7]. The major diseases that have plagued humanity such as smallpox, influenza, tuberculosis, malaria, measles and cholera have all evolved from zoonotic infections, which shows the effectiveness in humans of virulence traits evolving over transmission of anthropozoonoses [6,7,8]. Human mutualist and anthropozoonotic agents will not be considered here except for those that are primarily environmental bacteria. 

Besides human and other mammals’ microbiota, the environment is a huge reservoir for human pathogens. Water-, soil- and airborne pathogens, whether strict or opportunistic, represent major sources of human infections, either directly or when mediated by vectors such as food and medical devices. For instance, waterborne microbial diseases remain the leading cause of death worldwide with expanding spectrum and increasing incidence [10]. The water-associated infectious risk is greatest for water in the close environment of human beings, such as drinking water, domestic water, recreational water and water used in healthcare. Besides the main waterborne ‘true’ pathogens, bacteria from aquatic habitats are frequently involved in opportunistic infections and HAIs [11]. 

**Table 1:** Synthetic table showing the major characteristics of the natural behavior of selected environmental OBP. Data were extracted from the references cited in the review.

Environmental OBP	Habitat/ natural host	Lifestyle	Relationships with cells	Pathogenicity for humans
***Acetic Acid Bacteria***	Food	Food processing	Extracellular	HAI
	Fruits, flowers	Free living		Diverse mild infections in ID and CF
	Midgut, salivary glands of flying insects	Symbiotic		Bacteremia
				Chronic granulomatous disease
***Aeromonas* spp.**	Freshwater, chlorinated water	Free living	Extracellular	Diarrhea
	Polluted soils	Pathogen for fish, amphibian and mollusk		Wound infections
	Nematodes			Bacteremia
	Mosquitoes			
	Leeches, mollusks			
	Fish, Amphibians, Crustacean			
***Agrobacterium radiobacter / Agrobacterium tumefaciens***	Rhizosphere	Free living	Extracellular	HAI
	Plants	Phytopathogenic	Plant transformation	Diverse mild infections in ID and CF
				Bacteremia
***Burkholderia cepacia* complex**	Soil	Free living	Extracellular	Infections in CF (ET-12 epidemic clone)
	Rhizosphere	Plant-growth promoting	Facultative intracellular (plant, macrophage)	Chronic granulomatous disease
	Plant	Phytopathogenic		
	Amoeba			
	- *Acanthamoeba*			
***Chromobacterium violaceum***	Water and soil	Free living	Extracellular	Serious or fatal infections in ID and children
	in tropical and subtropical ecosystems			
***Legionella pneumophila***	Fresh water, chlorinated water	Free living	Facultative	Serious or fatal Pneumonia (Legionellosis)
	Amoeba	Amoeba-associated	Intracellular	
	- *Acanthamoeba*			
	- *Naegleria*			
	- *Echinamoeba*			
	- Hartmannella…			
	Ciliata:			
	- *Tetrahymena*…			
***Ochrobactrum***	Soil and polluted soils	Free living	Extracellular	HAI
	Rhizosphere	Dixeny with nematodes		Diverse mild infections in ID and CF
	Plants	Plant-growth promoter		Bacteremia
	Insects	Nodule formation in plant		
	Nematodes			
***Pseudomonas aeruginosa***	Fresh and Sea water	Free living	Extracellular	HAI
	Chlorinated water	Amoeba-associated	Facultative intracellular in amoeba	Wound infections
	Water distribution systems (hospital, domestic)			Burn infections
	Pharmaceutical water and antiseptics			
	Wastewater			
	Terrestrial wet ecosystems			
	Polluted soils			
	Rhizosphere			
	Amoeba			
	- *Acanthamoeba*			
***Photorhabdus luminescens***	*Heterorhabditis indica*	Symbiosis	Extracellular	No
***Photorhabdus asymbiotica***	Unknown	Non symbiotic	Facultative intracellular in macrophage	Serious soft tissue infections
				Bacteremia
***Serratia marcescens***	Plant (phloem)	Free living	Extracellular	HAI
	Insect	Phytopathogen		Ocular infections
		Squash bug (*Anisa tristis*)		
***Stenotrophomonas maltophilia***	Natural water	Free living	Extracellular	HAI
	Water distribution systems (hospital, domestic)	Plant-growth promoter		Infection in CF
	Pharmaceutical water and antiseptics			
	Wastewater			
	Rhizosphere			
	Deep-sea invertebrates			
	Food			
	Reptiles, mammals			

However, reducing the environment to 3 general elements (water, air and soil), as opposed to the human niche, leads to the conceptual error of medical microbiology, which is to consider the environment as an abstract entity merely surrounding human beings. In fact, our environments are multiple and essentially biotic, and environmental OBPs naturally have a community lifestyle associated with various other organisms, which are called shelter organisms [12]. In both water and soil, bacteria are sheltered by invertebrates, plants and protozoa, which are recognized as hotspots for genetic exchanges and the emergence of pathogens [12,13]. Given the current and ancient predominance of protozoa, plants and invertebrates, it is likely that bacterial interactions with shelter organisms are not only a present source of human pathogens but have also shaped bacterial evolution [14]. Bacteria sheltered by invertebrates, protozoa and plants are equipped with factors that overcome the innate defenses of their hosts. These factors might secondarily be useful for the adaptation of OBPs to human beings and might also further the spread of novel virulence factors into existing mutualist or pathogenic bacteria [14]. Hence, environmental hotspots of emergence and pre-adaptation, acting as genetic melting pots, are referred to as ‘nurseries’ for human OBPs [15,16,17]. 

### 2.1. Rhizosphere

In terrestrial ecosystems, the rhizosphere, which is the zone of adherence of soil to plant roots, is one of these hotspots because the biomass and activity of microbes are enhanced as a result of exudation from roots of compounds such as organic acids, sugars, amino acids and vitamins [17]. The genera *Agrobacterium* [18], *Burkholderia* [19], *Enterobacter* and/*or Pantoea* [20], *Herbaspirillum* [21,22,23], *Ochrobactrum* [24], *Pseudomonas* [25] and *Stenotrophomonas* [26], among others, entertain bivalent interactions with both plants and human tissues. Except for the notorious phytopathogen *Agrobacterium* [18] and the opportunistic plant pathogen *P. aeruginosa* [25], most of these rhizobacteria promote plant growth by various mechanisms, including antagonism against phytopathogens.

The fact that many bacteria in the rhizosphere are antibiotic producers could explain the frequent detection of bacteria with multiple antibiotic resistances in this micro-environment [17]. Moreover, several members of these genera show a common ability to degrade environmental pollutants [27,28]. This particular capacity to degrade xenobiotics is related to an extensive enzymatic arsenal that could play a role in the resistance to both cellular toxic products, such as free radicals [29] and antibiotics [30] observed in the clinical strains. In addition to xenobiotic-degradation enzymes, transporter pumps that push out xenobiotic molecules, including antibiotics, from inside bacterial cells have been widely detected in bacterial genomes and particularly in plant-associated bacteria [28]. Transporter overexpression, due to mutations in regulatory genes, can be easily selected using antibiotics and antiseptics, but also chemical compounds used for housekeeping, farming, and food processing [31]. Recently, two-component signal transduction systems were shown to control the expression of xenobiotic transporters that are transiently induced by environmental stimuli, such as low pH and osmotic changes, as encountered in both environment and infection sites [31].

The importance of xenobiotic transporters revealed by genomics and post-genomics suggests that xenobiotic resistance is not their natural function but that they probably have physiological substrates to transport [32]. Therefore, besides their natural function, versatile secondary functions, including the efflux of xenobiotics, became a ‘welcomed’ side effect. It has been hypothesized that bacterial virulence could be a by-product of xenobiotic transportation. Although this has not been studied in rhizosphere bacteria, recent studies on *Salmonella enterica* have shown that the xenobiotic transporter not only confers antimicrobial resistance but also contributes to virulence [33]. Reasons evoked are the capacity to transport secreted proteins necessary to establish virulence, such as toxins, the capacity to efflux antibacterial substances produced by the host and the capacity to transport factors effecting bacterial regulatory functions within the host, like signal transduction substances [31]. 

In addition to xenobiotic transporters, the lifestyle of bacteria in the rhizosphere predisposes them to become OBPs, thanks to several properties: production of bio-surfactants, competition for nutrients and minerals and degradation of pathogenicity factors naturally produced by phytopathogenic bacteria, which could be similar to those produced by the human immune cells. It has been suggested that plant response to bacterial toxins, flagella and lipopolysaccharide (LPS) resembles in many ways mammalian innate immunity [17]. Consequently, interactions with plant roots might pave the way for bacterial adaptation to mammalian and human cells. 

### 2.2. Protozoa

Amoebae normally feed on bacteria, but Rowbotham in an initial report on Legionnaires' disease noted the capacity of *Legionella pneumophila* to survive and multiply within amoeba. This finding was linked to the fact that macrophages, the natural human targets of *legionellae*, are indeed amoeboid cells, despite their evolutionary distance from amoeba [34]. It was then suggested that adaptation to amoebae served as a pre-adaptation stage to the macrophage internal environment, an important step in the process of becoming a human pathogen. Since these observations, molecular evidence has shown the similarities in the intracellular infections of macrophages and protozoa by *L. pneumophila* as well as similarities in the lifecycles of the bacteria within amoeba and macrophages [15]. Amoebae are now known to serve as a reservoir for various other pathogenic bacteria, including the OBPs *Escherichia coli*, *Burkholderia cepacia*, *Ralstonia pickettii*, *Listeria monocytogenes*, *Mycobacterium* spp. and *P. aeruginosa* [15]. Moreover, the amoeba operates as a ‘Trojan horse’, introducing the pre-adapted bacterium into the human host [35,36]. Another teleological metaphor is proposed by Schmitz-Esser *et al.* [37]: amoeba serves as a ‘training ground’ for bacteria that would acquire the capacity to live in a human organism, should they meet one. This strategy has also been observed for *Mycobacterium avium*, an opportunistic environmental pathogen for children and patients with AIDS; the co-culture of amoebae with *M.avium* enhanced the latter’s virulence, particularly its motility and ability to cross the murine intestinal epithelium [38]. Moreover, an enhanced resistance to antimicrobial agents, to biocides and to cold was observed [36]. The resistance of the amoeba-associated bacteria to cold suggests that amoebae serve as bacterial reservoirs at low temperatures in the environment but also on human body surfaces, such as nasal mucosa; subsequent lysis of the amoeba at higher temperature results in the dispersal of the bacteria, for example in the lower respiratory tract [15]. Virulence enhanced by amoebae is not limited to intra-amoebal or intracellular bacteria, amoeba-bacterium interactions in extracellular contexts occur as well. For instance, *Acanthamoeba castellanii* interacts with *Vibrio parahaemolyticus* without any engulfment, but rather by secreting a substance that promotes the survival of the bacterium in co-culture [39]. Free-living amoebae have also been described as melting pots for bacterial evolution, fostering horizontal genetic transfers (HGTs). For example, *L. pneumophila* and *Protochlamydia amoebophila*, which both live in amoebae, share genes with a phylogenetically unrelated intracellular bacterium, *Rickettsia bellii* [13]. This suggests the co-existence of their ancestors in amoebae prior to diverging evolution toward different life-styles, in amoeba and occasionally in human cells for *L. pneumophila*, in tick cells only for *R. bellii* and in amoebae only for *P. amoebophila*.

### 2.3. Insects

Humans live in close association with a variety of insects and other arthropods, which are well known as vectors for human diseases, such as malaria, leishmaniasis and arbovirus infections. From a phylogenetic point of view, *Bacillus anthracis* and *Bacillus thuringensis,* devastating for mammals and insects respectively, are believed to have evolved from an insect-associated *Bacillus cereus*-like ancestor that belonged to so-called "pre-vertebrate" pathosphere [14]. Common mechanisms are shared by pathogenic bacteria in the infective processes against both human and insect hosts. These mechanisms include adhesion to the host, entry and invasion of the host, establishment, dissemination within the host, toxin production, avoidance of host immune responses and transmission. At the genomic level, pathogenicity islands from mammalian and insect pathogens show astonishing similarity, suggesting that there is conservation of the genes involved in human and insect pathogenesis [14]. Moreover, human and insect immunity are also similar in many ways [16]. The analogies between insect and mammalian immunities, as well as between insect- and mammalian-associated bacteria virulence traits, suggest insects may act as a host reservoir of pathogens pre-adapted to the human innate immune system and who may ‘jump’ to humans, creating novel diseases [16]. 

The example of Acetic Acid Bacteria (AAB) illustrates this fact. AAB oxidize alcohols or sugars, leading to the production of acetic acid. They are commonly associated with plants and have been used in industrial food processing throughout human history, especially to convert wine to vinegar and to produce tropical fermented products [40]. It has recently been found that AAB of the genera *Acetobacter*, *Gluconacetobacter*, *Gluconobacter*, *Asaia*, *Saccharibacter* and *Commensalibacter* establish natural symbiotic relationships with the midgut of flying insects such as fruit flies, mosquitoes, honey-bees and leafhoppers [41]. This midgut niche has an acidic pH, a selective factor that supports AAB growth. In addition, AAB possess the capacity to migrate from midgut to other organs such as reproductive organs or salivary glands and hence be transmitted by vertical and horizontal routes. They also play a major role in the regulation of the insect innate immune system. Taken together, the data indicate that AAB represent novel secondary symbionts of insects [41]. In parallel, AAB are emerging as human OBPs. The first report of human infection involving AAB was a case of peritonitis associated with *Asaia bogorensis* [42]. Since then, AAB have increasingly been reported as responsible for human infections, particularly in CF patients [43]. The ability to grow in acidic conditions, as observed in insect midgut, may confer on AAB a selective advantage in the abnormally acidified airways of CF patients [43]. Besides this common adaptation trait, analogies between insect and mammalian immunity and susceptibility to bacterial virulence factors as well as the intimate association of AAB with both humans (through food) and insects (through plants) might drive the evolution of these environmental bacteria toward becoming emerging insect symbionts and human OBPs. 

Some insect-associated bacteria can adapt to a larger range of hosts. *Serratia marcescens* displays adaptation to insects and plants but also to human beings. The jump from insects to plants has been observed for phytopathogenic bacteria. Intimate associations with plants predispose phytopathogens to frequent encounters with herbivorous insects that could evolve to become specific vectors for pathogens or alternative primary hosts for phytopathogenic bacteria [44]. *S. marcescens* is a phloem-resident pathogen that causes yellow vine disease of pumpkins and squashes transmitted by the squash bug, *Anasa tristis* [45]. Not only adapted to plants and insects, *S. marcescens* is also an emerging human pathogen that is predominantly involved in HAIs, particularly in neonatal intensive care units [46] and also implicated in a range of ocular infections [47]. One factor that may enable *S. marcescens* to thrive in a variety of different environments is its ability to colonize surfaces and form biofilms. Its multiple processes of surface colonization are quorum sensing-regulated, and include swarming motility, biofilm maturation and detachment in the environment. These quorum sensing-regulated properties allow *S. marcescens* to become a good colonizer of living surfaces in plants, insects and humans as well as on inanimate surfaces such as medical devices or corneal lenses [48]. Adaptation to one host could allow for pre-adaptation to another one, hence producing specialization in each host while widening the host range.

### 2.4. Worms

We will show below in detail (see chapter below: *Horizontal Genetic Transfers give evolution a boost*, example of *Photorhabdus luminescens*) that worms as well as insects and other invertebrates can be considered as shelters or ‘nurseries’ in which adaptation to the mammalian niches is prepared. Worms clearly belong to the so-called ‘pre-vertebrate pathosphere’.

The successful use of *Caenorhabditis elegans* as an infection model to understand microbe-host interactions and to study pathogenesis shows that common mechanisms of virulence are shared among the different kingdoms of life. Today, approximately 40 bacterial and fungal pathogens are known to harm *C. elegans*. The regulatory networks of bacterial pathogens seem to be conserved across multiple infection models suggesting that they co-evolved during the history of interactions between *Bacteria* and *Eucarya*. As a consequence, some of the bacterial regulators, such as quorum sensing molecules and two-component systems, interact with innate immune functions shared at the interkingdom level. The *C. elegans* model is particularly efficient in detecting virulence traits in OBPs because virulence in strict pathogens is more often host-specific and cannot be universally modeled in the nematode infection system [49].

## 3. From Environment to Man: Lessons from OBP Genomics

Despite the existence of shelter organisms acting as relays throughout adaptation to man, growth of bacteria of the same species or of the same genus in both environmental and human niches is a major challenge. Bacterial genome analysis and comparative genomics reveal common traits in genome evolution, leading to pathogenicity for human beings and/or to emergence of human OBPs.

Comparative genomics in bacteria have clearly shown that differences in genome size reflect variations due to the acquisition and loss of DNA portions, a common mechanism of bacterial genome evolution [50]. Aside from gene acquisition and loss, modifications of gene order can occur, and synteny is often not maintained among related genomes. In recent critical commentaries on genetics, James Shapiro [51] described genome restructuring as ‘cellular virtuosity in rewriting […] DNA’. Genome fluidity concerns mainly the flexible gene pool, *i.e.*, an assortment of genetic information, enabling a bacterium to adapt to special conditions, such as those involved in the colonization of new ecological niches, symbiosis, host interaction and pathogenicity. In contrast, the core bacterial genome ensuring basic functions remain common to several strains or species [50,52]. Consequently, genome organization, notably size and dynamics of the flexible genome, reflects bacterial lifestyle [52].

### 3.1. A Large and Fluid Genome Is the Key to Bacterial Versatility

Some species of environmental bacteria have an extraordinary range of behaviors and activities, such as free life in water, breakdown of pollutants, production of original biomolecules, beneficial effects for plant growth and health, phytopathogenic effects, association with animals and also multi-drug resistance and human pathogenicity. The wide range of activities of versatile life-style bacteria involves adaptation to various niches, which is supported by a larger genome than phylogenetically related specialized bacteria [52]. The content of 115 bacterial and archeal genomes analyzed by Konstatinidis and Tiedje [53] showed that larger genomes (>6 Mb) accumulate secondary metabolism, and, to a smaller degree, energy conversion-related genes. They also display an obvious increase in regulatory genes to successfully control the extensive metabolic repertoire they express under different growth conditions. In the Konstatinidis and Tiedje study [53], all species with genome size >6 Mb lived in very diverse environments with a great range of substrates for energy production. In particular, free life in water or soil provides the bacterium with varied but scarce sources of nutriments [53]. Bacteria with large genomes are frequent in nature. In spite of the bias induced by preferentially sequencing bacteria with small genomes, which are also frequently pathogenic for humans [54], large genomes of more than 6 Mb are widely represented. Consequently, one may consider that such genomes are ecologically successful with little penalty for the slow growth generally related to high genome size [53,55]. 

*Chromobacterium violaceum* is a free-living microorganism that populates the soil and water in tropical areas around the world [56], and is abundant in the Rio Negro in Amazonia. *C. violaceum* is mainly a saprophyte but is also an OBP for infants and immunocompromised patients. However, the fact that the Rio Negro is the source of drinking water for the population living around it, without there being widespread infection, indicates the low infectivity of this organism. While *C. violaceum* infections are scarce, their fatality rate is high [29]. Total genome sequencing of *C. violacaeum* reveals that its adaptability and versatility depend on genes specifically related to interaction with and response to environmental variations. Most represented among encoded proteins are those involved in versatile pathways for energy generation, transport, stress adaptation, motility systems and quorum sensing for control of inducible systems. Of note: *C. violaceum* produces an exceptional range of enzymes including paraquat-inducible proteins, drug and heavy-metal resistance proteins, multiple chitinases, and proteins for the detoxification of xenobiotics [56]. Violacein is the major secondary metabolite of *C. violaceum* and responsible for the pigmentation of the bacterium. The violacein pigment, which has already been used as a therapeutic compound for dermatological purposes, also exhibits antimicrobial activity against *Mycobacterium tuberculosis*, *Trypanosoma cruzi*, and *Leishmania* sp. and is reported to have other bactericidal, antiviral, and anticancer properties [56]. The Brazilian consortium credited with sequencing the genome of *C. violaceum* expresses hopes that their work will not only benefit biotechnological and pharmaceutical industries in the developing world, but will also provide “a further stimulus to the preservation of the precious ecosystems where these organisms are found.” The realization that a bacterium can belong to our biodiversity heritage is infrequent enough to be noted.

Regarding pathogenicity in humans, type III secretory system (T3SS) components similar to those in *Salmonella enterica* serovar Typhimurium and *Yersinia pestis* have been found under an incomplete form in *C. violaceum*. The similarity of this T3SS with those of human pathogens suggests that it contributes to human infection, its incompletion explaining that human infections are very infrequent and limited to predisposed patients [56]. Besides T3SSs, which are typical and specific virulence factors for humans and animals, several other genes are believed to be involved in pathogenicity. *C. violaceum* possesses non-specific virulence factors such as pili, flagella, LPS and peptidoglycan [29,56]. However, most bacteria display these factors without being pathogenic. Rather than true pathogenic traits, they could be considered as versatile factors that also support the adaptation of *C. violaceum* to its environmental niches. The closest similarity between the corresponding genes and their homologs in pathogenic bacteria was found for *Neisseria meningitidis* and *P. aeruginosa*. This has been evoked to explain the pathogenicity of *C. violaceum* [29], but is not fully convincing because *N. meningitidis* and *P. aeruginosa* are phylogenetically *Gammaproteobacteria*, related to *C. violaceum*. This relatedness alone could explain the high similarity level obtained with the corresponding genes in *N. meningitidis* and *P. aeruginosa*. The relationships between environmental specific functions and pathogenesis are better illustrated by the detoxifying capacities of *C. violaceum*. A part of the arsenal for detoxification of environmental xenobiotics revealed in *C. violaceum* genome contributes to reduce harmful free radicals. This function could be considered as an environmental pre-adaptation for bacterial evasion of the human host immune system by resistance to the free radicals produced by phagocytes. Clinical isolates show a 30% higher superoxide dismutase activity and a five-fold higher catalase activity than the corresponding activities observed in soil strains [29]. This observation suggests that strains exhibiting a powerful reduction of free radicals are successful in the human niche. As another example, violacein presents different properties (see above), all linked to its generalized cytotoxic capacity [29], which could play a part in the infectious process. 

The genome size of *C. violaceum* is about 4.7 Mb, which is not so large considering its metabolic versatility. This is also true for the OBP *Ochrobactrum anthropi*, whose genome size varies widely from one strain to another among the 9 studied [57]. The genome size variability within a species suggests that the functional versatility observed for a given strain is amplified at the population level. For these bacteria, the pangenome reflects the variability of the flexible genome and the overall coding capacities of the species.

Besides the size, genomic fluidity resulting in rearrangement events is another way to increase variability, and thus improve versatility, at the population level. Ogier *et al*. [58] carried out an interesting study of comparative genomics on *Photorhabdus luminescens* variants obtained from their natural hosts. They followed short-term genomic rearrangements during genome speciation, thereby showing the role of intragenomic rearrangements in the processes responsible for bacterial genome diversification and evolution. Due to the modular organization of the chromosome, rearrangements lead to a combinatorial process rather than to a random patchwork. The fluid nature of genomes can be seen as ‘natural genetic engineering systems’ that facilitate the evolutionary rewriting of information [51]. In particular, rearrangements maximize the chances of success by using combinatorial processes based on basal functions while maintaining a reasonable genome size [51].

Genome fluidity in a species is easily detected by the use of DNA macrorestriction methods. For instance, RFLP followed by Pulsed Field Gel Electrophoresis (PFGE) shows highly diverse migration profiles for genomes subjected to extensive rearrangements [59]. PFGE is currently one of the most performant methods in molecular epidemiology of infectious diseases, particularly when infections are caused by *P. aeruginosa*, enterobacteria or other OBPs [59,60]. In particular, PFGE is more discriminative than sequence-based methods such as multilocus typing, which indicates that genome rearrangement is the principal and/or the most rapid mode of evolution in these bacteria.

In summary, fluidity by intragenomic rearrangement is a major process of diversification and evolution of the OBPs genomes. Another cause of genome fluidity in bacteria is the acquisition of foreign DNA by horizontal genetic transfer (HGT).

### 3.2. Horizontal Genetic Transfers Give Evolution a Boost

Large bacterial genome size has been related to the population size and the rate of HGT [61]. It has been shown that free-living species of large population size accumulate insertion/deletion and rearrangements at much higher frequencies than host-dependant bacteria that encounter a bottleneck in population size. In the latter, the influence of HGT is negligible and evolution occurs mainly by nucleotide substitution [62]. 

Expansions and contractions in the genomic repertoire mainly affect genes involved in environmental interactions. Duplications and HGTs allow a gain of information more rapidly than punctual mutations. These mechanisms of high-rate evolution enable rapid responses to alterations in the environmental conditions subjecting the bacterium to strong selective pressure [61]. If environmental conditions vary frequently, acquired characters will be conserved in large genomes, expanding and diversifying the metabolic and regulatory capacities of the bacterial cell. 

Comparing the genomes of closely related strains or species with different lifestyles could help to understand the role of HGT in the emergence of OBPs. The genus *Photorhabdus* provides a valuable example [63]. *P. luminescens*, the only terrestrial bacterium exhibiting bioluminescence, lives in symbiosis with soil entomopathogenic nematodes. This bipartite natural system is currently used for the biological control of crop pests. Some *Photorhabdus* strains belonging to the species *Photorhabdus asymbiotica* provoke invasive soft tissue infections and bacteremia in humans. *P. asymbiotica* has a smaller genome than that of the insect pathogen. The difference in size is about 600 kb, which is to say about 600 genes and 10% of the total genome size. Moreover, one megabase of DNA appears unique to each strain sequenced [63]. Contrasting with its smaller genome, *P. asymbiotica* is the only *Photorhabdus* to carry plasmids [63]. The plasmid pAU1 reveals a wide array of transposons similar to those found in genome and plasmids of *Y. pestis*. The genomes of *P. asymbiotica* and *P. luminescens* show strong synteny across much of their length. However, there are several large-scale inversions in the central regions of the chromosomes associated with numerous transposons and repeat sequences. The genome of *P. luminescens* has been previously shown as highly plastic with genomic variations occurring in clonal populations. The phenotypic consequences of these genomic changes are cryptic but a role in adaptation to environmental conditions is suggested [64].

Differences in gene content between *P. luminescens* and *P. asymbiotica* consist in several genes coding for human host-specific virulence factors, notably genes coding for T3SSs. Effectors of T3SS that inhibit phagocytosis of *P. luminescens* following its translocation into insect hemocytes were replaced in *P. asymbiotica* by the ExoU-like effector. ExoU is a *P. aeruginosa* toxin with a phospholipase activity that disrupts human macrophages and has also been implicated in the T3SS-mediated killing of amoebae that graze *P. aeruginosa* biofilms [65]. It is noteworthy that a human pathogen-associated virulence factor, namely a cell invasion factor translocated via the T3SS, was also detected in *P. asymbiotica*. This gene, named *sopB*, is important in ‘directing traffic’ in the early stages of *Salmonella enterica* serovar Typhimurium entry into host cells by modulating interaction of *Salmonella*-containing vacuoles with the endocytic system [63,66]. 

Traits that enable *P. asymbiotica* to resist the fast-acting human innate immune response are also detected. Indeed, a small protein with homology to the attachment invasion locus protein Ail, that gives *Yersinia pestis* resistance to human complement, is secreted at 37 °C by *P. asymbiotica* but not at 30 °C [63]. Other genes found in *P. asymbiotica*, but not in other *Photorhabdus* strains, display homologies with various genes of human pathogens such as *Vibrio cholerae*, *V. parahaemolyticus*, *Y. pestis*, *P. aeruginosa* and *Bordetella parapertussis* [66]. Comparative genomics between *P. luminescens* and *Yersinia enterocolitica* identified several common loci representing ancestral clusters of genes important in pathogenesis. These might have evolved during the association of the two bacteria with invertebrates and then adapted to more recent pathologies in mammals. Examples are yersiniabactin, quorum sensing-like regulators, or the urease operon [67]. *Y. enterocolitica*, similar to *Y. pestis*, which has developed a strategy to infect and proliferate in insects. *Y. pestis* probably met *P. asymbiotica* in the ‘pre-vertebrate pathosphere’ as *Y. enterocolitica* met *P. luminescens*. This suggests that the emergence of *P. asymbiotica* as an OBP is mainly due to HGT from other human pathogens. The emergence of *P. asymbiotica* as a human pathogen is an emblematic example that reinforces the hypothesis of an environmental ‘nursery’ for human OBPs, detailed above: insect and nematode immune systems as well as potential relationships with amoeba make the bacterium able to evolve from resisting invertebrate immune system and/or amoeba grazing to resisting the human immune system by taking refuge in macrophages in the early stages of infection.

HGT events are obvious in most OBPs but are probably not sufficient to spawn the emergence of OBP behavior as exemplified by the case of *Burkholderia cenocepacia*. The strain J2315 of *B. cenocepacia* belongs to an epidemic lineage named Edinburgh–Toronto (ET-12) associated to CF patients whereas other strains of the species are more versatile and are found in soil, plant and man. Its 8.06-Mb genome comprises three circular chromosomes and a plasmid and encodes a broad array of functions typical of this metabolically versatile genus, as well as numerous virulence and drug resistance functions. Comparative analysis revealed that 21% of its genome is unique and highly specialized in comparison to other strains of *B. cenocepacia* [68]. Among J2315 specific genes, a new collagen-binding trimeric autotransporter adhesin with no bacterial orthologs was found. This gene has a role in cellular adhesion and virulence [69] and was acquired by HGT, although the parental donor remains unknown. 

HGTs undoubtedly promote and contribute to the success of environmental bacteria as OBPs but other signs of genomic evolution were detected in the J2315 strain. For instance, pseudogenes indicative of genomic reduction are detected, suggesting that both gain and loss of functions have occurred in this OBP, specialized to persist in the CF lung [68]. 

### 3.3. Genome Reduction: No Turning Back

Gene loss is a general mechanism of bacterial evolution that avoids genetic redundancy and helps maintain a reasonable genome size in bacteria. Genome reduction is related to close association with host cells as observed for intracellular bacteria. Consequently, genomic reduction is a main force behind the evolution of parasitic and/or intracellular bacteria and the emergence of strict pathogens from OBPs [61,70,71]. A recent study compiling current genomics data confirmed that the evolution of specialized bacteria, including pathogenic bacteria, consists mainly of gene losses [72]. Moreover, extreme genome decay is often accompanied by a low GC% content [73]. A bacterium that establishes intracellular relationships is surrounded by the host cell’s metabolic products. Some bacterial metabolic pathways, redundant with those of the host cell, become non-essential and will be subject to genetic degradation due to the loss of selective pressure that acts upon them [74]. The closer the relationships the more genetic decay occurs. For example, the obligatory intracellular bacteria of the genus *Rickettsia* no longer have genes for amino acid and nucleotide biosynthesis. In the *Alphaproteobacteria* super-class, genome comparison of the facultative intracellular bacteria *Bartonella henselae* and rickettsiae shows that obligatory parasites display about 1,000 genes less than facultative ones. This repertoire of 1,000 genes probably represents the differences in metabolic needs between facultative and obligatory intracellular bacteria [75]. To take a more extreme example of endosymbiotism, the genome of a mitochondrion, which is an ancient alphaproteobacterium, displays about 800 genes less than *Rickettsia prowazekii* [74]. The genomic decay in intracellular bacteria is not only related to the change in selective pressure when the bacterium is associated with a narrow niche, but also to a change in population structure. Narrow niches-associated bacteria encounter a population bottleneck that leads to the emergence of small clonal populations isolated from foreign genetic information. In these conditions, slightly deleterious mutations accumulate without being compensated for by transfer of new genes [76]. The most radical mechanism of genomic reduction observed during adaptation to a narrow niche is loss of plasmids. Indeed, plasmids can impair bacterial fitness particularly by slowing down growth rate in the absence of environmental selective pressure [77]. For instance, none of the animal cell-associated alphaproteobacteria display plasmids while alphaproteobacteria living in soil or in the rhizosphere such as *Rhizobiaceae* carry various plasmids and often megaplasmids of more than 1 Mb [61]. 

Besides loss of plasmids, degradation and deletion of coding sequences in the chromosome also occur. Genomics provides examples of gradual degradation of gene contents with intermediates ranging from intact ORFs to complete gene disappearance through transcribed split ORFs, further split ORFs no longer transcribed and fully decayed but still recognizable ORFs [71]. All these intermediates are recognized as genomic scars, named pseudogenes. The gradual gene degradation is particularly obvious in comparative genomics of rickettsiae [71] but also occurred in the genome of other human pathogens such as *Mycobacterium leprae*, *S. enterica* serovar Typhi and *Y. pestis* [61]. More generally, the presence of a large number of pseudogenes is significant of genome reduction related to niche specialization. Large deletions due to accidents during recombination between repeated sequences are another mechanism of genome reduction [78]. Such deletions are detected in comparative genomics by the loss of a block of genes located between two repeated sequence copies as well as by the loss or the disturbance of one repeated sequence copy [76,78].

Comparative genomics in the super-class *Alphaproteobacteria* suggest that *Rickettsiaceae* lost 2,135 genes during their evolution to an intracellular lifestyle [79]. The comparison of 11 *Rickettsia* spp genomes shows that the differences in gene repertoires are mainly the result of differential gene losses from the rickettsial ancestor. The different genomic repertoires seem to play important roles in the adaptation of *Rickettsia* spp. to their various hosts, greater gene loss and subsequently smaller genomes being related to restricted host range. This diversity also appears to be crucial for the emergence of new species [79].

A longstanding belief was that ‘true’ pathogens are highly adapted to their hosts. The current vision shows pathogens as highly specialized in virulent behavior against their hosts. Georgiades *et al*. [54] recently proposed a scenario where pathogenicity of ‘true’ pathogens appears as an ‘unadapted’ behavior. Non-specialized bacteria such as OBPs enjoy a community lifestyle, which allows them to exchange genes resulting in an increased genome complexity whereas specialization results from a purifying selection [80]. At some point in their evolutionary history, the non-specialized bacteria can become specialized organisms in different niches. Subsequently, gene exchanges decrease and the gene repertoires undergo changes by differential reduction. The specialization of organisms results in gene loss. Regulatory gene loss and the resulting deregulation eventually leads to uncontrolled multiplication that destroys the ecosystem, *i.e.*, host cells and tissues. In this evolutionary dead end scenario, the specialized bacteria appears as ‘unadapted’ [81].

We showed that pre-adaptation of environmental bacteria to pathogenicity for humans is related to genomic traits, while emergence of OBPs is the result of genome dynamics. The genera *Pseudomonas*, *Aeromonas* and *Ochrobactrum* will provide us with valuable examples of opportunistic behavior associated to particular genomic structure and evolution, with each of these OBPs displaying a refined association of genomic traits that shed light on their particular OBP behavior.

## 4. Pseudomonas aeruginosa, the *Swiss Army Knife*

*P. aeruginosa* is a major environmental-borne human OBP. It is able to live in a wide range of environments: aquatic and wet ecosystems such as rivers [82], open ocean [83], wastewater [84], and various terrestrial environments including rhizosphere, soil from agricultural lands and hydrocarbon-polluted sites [85]. *P. aeruginosa* is the third cause of HAIs, its resistance to antibiotics [86] and its ability to thrive in wet environments have been related to its nosocomial success. Aside from HAIs, *P. aeruginosa* causes a wide range of acute and chronic human infections in predisposing situations such as chronic wounds, burn wounds and chronic obstructive pulmonary disease, particularly in CF patients. The colonization of this broad spectrum of habitats is enabled by metabolic versatility and a high potential for adaptation to changing environmental conditions [87]. *P. aeruginosa* can use a variety of carbon sources, has minimal nutrient requirements, utilizes nitrogen for anaerobic respiration in addition to its preferential aerobic behavior and grows at temperatures up to 42 °C. This versatility is the result of genomic and evolutionary mechanisms leading to a highly flexible repertoire of genes that ensure survival in diverse environments and expansion of niches. 

The first sequenced genome in the species *P. aeruginosa* [88] revealed genomic traits that can account for its phenotypic versatility. First, the genome of *P. aeruginosa* is larger than that of most bacteria that cause human infections. Indeed, most specific human pathogens have undergone a reduction of their genome during their adaptation to a narrow niche or to intracellular existence [89,90]. The second information derived from *P. aeruginosa* sequencing is that it contains genes families encoding very diverse functions in contrast to other large bacterial genomes made large by gene duplication events rather than functional diversity. In particular, the *P. aeruginosa* genome contains a disproportionately large number of genes encoding outer membrane proteins involved in relationships with biotic and abiotic environments *i.e.*, adhesion, motility, efflux, export and sensing by two-component systems. Consistent with the bacterium’s metabolic versatility, *P. aeruginosa* genome comprises a large number of genes encoding transport systems and enzymes involved in nutrient uptake and metabolism [91]. Moreover, 8.4% of *P. aeruginosa* genes are involved in regulation, which is one the highest percentages of regulatory genes observed in bacterial genomes [88].

Initially compared by low-resolution physical mapping techniques [92,93], *P. aeruginosa* genomes can now be compared by DNA sequencing technologies [91,94]. Despite the fact that *P. aeruginosa* thrive in highly diverse ecological niches, complete genome sequences are available or in progress mainly for *P. aeruginosa* isolates from human origin (28 strains among 29). Five genomes are currently fully annotated. The first complete genome sequencing was performed for strain PAO1 [88] derived from an Australian wound isolate from the 1950s. The PAO1 strain is still the major reference for genetic and functional studies on *P. aeruginosa.* Later the genome of ExoU-positive strain PA14, a clinical isolate showing higher virulence than PAO1, was published [95]. The third sequence was that of strain LESB58 [96], the so-called ‘Liverpool epidemic strain’, found to be highly virulent and transmissible among CF patients. The LESB58 strain was noted for its capacity to cause infections even in non-CF human hosts [97,98] and for hosting previously unknown accessory elements [96]. Finally, the fourth strain to be sequenced was PA7, a clinical isolate from Argentina with an unusual antimicrobial resistance profile (resistant to many third generation antibiotics). The PA7 genome displays only 93.5% identity in the core genome with the other sequenced strains, confirming the strain PA7 to be a taxonomic outlier within the species *P. aeruginosa* [99]*.* To date, *P. aeruginosa* M18 that originate from the rhizosphere and used for biocontrol is the sole environmental strain to be sequenced and analyzed in detail [25]. Five almost complete genome sequences (last checked on January 25th, 2012) listed in the National Center for Biotechnology Information (NCBI) are also available for comparative genomics (Table 2). Strain PA2192 [100] is an isolate from a chronically infected CF-patient in Boston and has undergone significant phenotypic adaptation, characteristic of a majority of CF isolates, including conversion to mucoidy (due to a nonsense mutation in its *mucA* gene), production of lipopolysaccharide devoid of O-side chains, and lack of motility. Strain C3719 [100], the so-called Manchester epidemic strain, has been associated with enhanced virulence and transmissibility [101]. With the exception of mucoid conversion, it has also undergone similar adaptations associated with chronic CF isolates [101]. The three last genomes in progress are: PACS2 [100], 39016 [102] and PAb1 [103]. Nineteen other projects of *P. aeruginosa* genome sequencing are deposited in the NCBI (http://www.ncbi.nlm.nih.gov/bioproject).

**Table 2:** Genomic features of sequenced *P. aeruginosa* strains adapted from KLOCKGETHER *et al*. [104] and completed using NCBI genome data.

Strain	PAO1	UCBPP PA14	PA7	LESB58	PACS2	PA2192	C3719	39016	PAb1	M18
Accession Number	NC_002516	NC_008463	NC_009656	NC_011770	NZ_AAQW	NZ_AAKW	NZ_AAKV	NZ_AEEX	NZ_ABKZ	CP002496
Source	Wound	Clinical	Clinical	CF-patient	Clinical	CF-patient	CF-patient	Keratitis	Frost bite	Rhizophere
Genome size (Mbp)	6.264	6.538	6.588	6.602	6.492	6.905	6.222	6.667	6.078	6,327
GC-content (%)	66.6	66.3	66.5	66.5	66	66.2	66.5	66	66	66.5
No. of ORFs	5570	5892	6286	5925	5676	6191	5578	6401	5943	5684
% of coding sequences	88.9	90.1	95.4	89.7	87.4	89.6	89.6	96	89	89
Pseudogenes	5	0	8	34	0	2	3	9	0	6

### 4.1. A Mosaic of Core and Accessory Genes

The core genome of *P. aeruginosa* strains from both clinical and environmental sources represents about 90% of the total genome [25,91]. Comparative genomic analysis of PAO1, PA14, PACS2, C3719 and PA2192 revealed that 5,021 genes are shared by all five genomes. More than 90% of these genes display a low average of nucleotide divergence with at least 98% identity in sequence [100]. As for most bacteria, core genome encodes housekeeping proteins involved in central metabolism. Surprisingly, pathogenic factors are encoded by the core genome of *P. aeruginosa*, *i.e.*, shared by all strains irrespective of their origin. For example, a microarray-based comparison of 18 different *P. aeruginosa* strains found that 97% of the 267 examined PAO1 virulence-related genes were conserved across all strains [105]. A set of 980 genes, classically considered as non-essential because they are not involved in regulation, motility and virulence, shows no sequence variation at all among 36 clinical isolates. This indicates that these genes encode factors of evolutionary importance for the lifestyle of this successful environmental bacterium and opportunistic pathogen [106]. At the phenotypic level, common traits between environmental and opportunistic lifestyles can be highlighted. For instance, the ability to form polysaccharide-encased, surface-attached communities, known as biofilms, and to resist protozoan predation by injecting cytotoxic effector through a T3SS into the cytosol of eukaryotic cells can support persistence of *P. aeruginosa* both in nature and in colonization/infection of human beings.

However within the species, the fact that genome size varies between 5.2 and 7 Mbp [107] suggests accessory genome variability. Plasticity of accessory genome in *P. aeruginosa* is responsible for intra- and inter-clonal genome diversities that are easily detected by macrorestriction and PFGE*.* Several strains sharing the same genotype by single nucleotide polymorphism analysis in housekeeping genes were found to have different macrorestriction profiles. This fact supported the hypothesis that changes in *P. aeruginosa* genomes occur at a higher rate in the accessory DNA segments than in the conserved core genome [59]. Consequently, macrorestriction is the more discriminative method to trace clonal strains, for instance during outbreaks [60,108]. 

The elements of the accessory genome appear as “foreign blocks,” acquired by HGTs from other species or genera. The acquired blocks are interspersed among the genes of the core genome. Therefore, the overall architecture of the *P. aeruginosa* chromosome is often described as a mosaic structure. The fluidity of the mosaic observed by comparative genomics suggested recombination events but some physically distant loci exist in fixed combinations of genotypes, suggesting that the free flow of genes did not occur at all loci of the *P. aeruginosa* genome [107]. 

The accessory genome is not randomly scattered throughout the core genome but formed by dispersed polymorphic strain-specific segments, flanked by conserved genes used as anchors. The elements of the accessory genome can be present in subgroups of the *P. aeruginosa* population or only in single strains [104,107,109]. Mathee *et al*. [100] defined strain-specific regions of genomic plasticity (RGPs) as blocks of at least four contiguous open reading frames (ORFs) that are not conserved across all five *P. aeruginosa* genomes analyzed. Within the core genome, these RGPs are specific loci that act as hotspots for the insertion of accessory genes. In particular, tRNA genes in the core genome are frequently targeted for the insertion of accessory genetic elements [110]. Together with plasmids, the RGPs form a major part of the accessory genome. RGPs often contain mobile DNA elements acquired and kept by the host strain, such as integrative and conjugative elements (ICEs), phages, transposons or insertion sequence elements (IS). A recent review by Kung *et al*. [91] describes the different types of accessory elements. Interestingly, some RGPs, named replacement elements, belong to the core genome. They correspond to loci encoding proteins such as O-antigens, pilin, pyoverdin, that display less than 70% identity between homologs, despite being shared by all strains in the species. These replacement elements are under diversifying selection [104].

The analysis of seven sequenced *P. aeruginosa* strains (PAO1, PA14, LESB58, PA7, PA2192, C3719, PACS2) reveals 79 distinct RGPs [104]. The comparison of RGP gene content among strains reveals the highly customized nature of the genomes of 5 pathogenic *P. aeruginosa* [100]. RGPs appear to be the source of specialized functions that probably allow the survival of a particular strain in its environmental niche. For instance, in strain PA2192, evolution of RGP29 was linked to the acquisition of new metabolic capacities, such as growth in environments rich in abietane diterpenoid resins that are produced by conifers. Besides this outstanding behavior, PA2192 also establishes persistent infections in CF patients [100]. Acquisition of new traits appears as an expansion of the repertoire of functions rather than a specialization; indeed new traits do not eliminate pre-existing ones. 

### 4.2. The Eclectic Specialist

Studies at the population level show that the spread of dominant clones in both clinical and environmental habitats is a general feature of *P. aeruginosa* [107]. In Wiehlmann’s study [107], genotyping of a collection of 240 strains isolated from diverse habitats and geographical origins revealed that 16 most common clones made up half of the strain panel and were found to be widespread in multiple habitats and locations. Major clones are just as versatile in their local habitat as the whole *P. aeruginosa* population. For instance, Pirnay *et al*. [111] demonstrated that the local *P. aeruginosa* community in a Belgian river was almost as diverse as the entire terrestrial population of *P. aeruginosa*. The Belgian river harbored clones that also belonged to a collection of 73 clinical and environmental isolates previously collected across the world [112]. Such large-scale population studies did not detect strains specialized to particular niches such as human hosts. However, some specialized populations have been described, particularly for *P. aeruginosa* strains associated to the respiratory tract of CF patients.

*P. aeruginosa* infections in CF are a paradigm of how environmental bacteria can conquer, adapt to and persist in an atypical habitat and successfully evade defense mechanisms and chemotherapy in a susceptible host [113]. Considering core genome based phylogeny, the strain LESB58 hypervirulent for CF patients is the closest relative to the rhizosphere strain M18 [25]. However, dominant epidemic clones have been associated to CF. They display a phenotype distinct from other *P. aeruginosa* isolates characterized by reduced levels of virulence factor secretion [114], O-antigen deficiency [115], changes in LPS fatty acylation [116], amino acid auxotrophy [117], non-flagellation [118] and mutator phenotype [119]. The production of alginate and the formation of biofilms within the lung habitat are increased [120]. The genetic basis for this outstanding phenotype is point mutations in structural or regulatory genes, like *mutS* [121], *mucA* [122], *rpoN* [123] and *lasR* [124]. 

In early stages of infection in CF patients, the population of *P. aeruginosa* is diverse and displays phenotypes comparable to those of environmental isolates [125]. In contrast, adapted dominant epidemic strains are often identified from patients chronically infected with *P. aeruginosa* [126,127]. However, the dominant clones are probably present in the diverse population at the early stages of the disease [127]. The microevolution of *P. aeruginosa* populations in CF airways could be driven by hypermutable strains [119]. Proteomic and transcriptomic analysis of hypermutator CF strains showed an expression of genes involved in microaerobic lifestyle created by mucus in the lungs of CFpatients [128]. Besides point mutations, specialization for CF airways is accompanied by reorganization of the *P. aeruginosa* genomic structure by acquisition or loss of variable genomic regions [129] and genome rearrangements such as highly frequent large chromosomal inversions [130]. 

The genome organization and functionality of *P. aeruginosa* support the Swiss-army knife metaphor. Genome fluidity, acquisition of new specific genes (such as genes allowing life with toxic resins in ecosystems with conifers) and expression of phenotypic traits (such as alginate production in CF patient airways) account for adaptation to very particular niches, while mosaicism of core and accessory genes conserve versatile traits ensuring life under all conditions.

## 5. *Aeromonas hydrophila*, Jack-of-all-trades, and Its Relatives

Aeromonads display a large spectrum of lifestyles from free-living freshwater bacteria to symbionts of a variety of blood feeder organisms, to opportunistic pathogens of fish, amphibians and human. The range of habitats is exceptionally wide, from hostile environments such as polluted or chlorinated water, to nematodes, insects, fish and mollusks, other animals and man [131]. The genus is characterized by a large genetic and taxonomic diversity, to date 24 species. Three of them, *Aeromonas caviae*, *A. hydrophila* and *Aeromonas veronii*, are responsible for more than 85% of animal and human infections, while another, *A. salmonicida*, is restrictively involved in furonculosis of fish [131]. Human infections are extremely diverse but the most frequent are skin and soft tissue infections (SSTI), bacteremia and diarrhea (approx 90% of aeromonoses, [131]). 

The complete genome sequence of *A. hydrophila* ATCC 7966^T^ uncovered a broad metabolic capability and considerable potential for virulence factors, confirming its status of emerging generalist opportunistic pathogen, and earning it the nickname of ‘Jack-of-all-Trades’ [132]. Further genome sequences of closely related species are complete (*Aeromonas veronii, Aeromonas salmonicida*) or in progress (*Aeromonas caviae*) and shed some light on genome evolution and adaptation related to diverse behaviors. We will hereafter focus on 3 closely related aeromonads: *A. hydrophila*, *A. veronii* and *A. salmonicida* as various examples of genome evolution and adaptation. 

### 5.1. Water and Other ‘Nurseries’

Aeromonads are found primarily in freshwater where virtually all species of the genus may be recovered. They have a sympatric lifestyle that favors HGTs and high genetic diversity [54]. Water is the most frequent source of human infection, (i) either for SSTIs, mainly caused by *A. hydrophila* and *A. veronii*, usually at a rare frequency, except in case of natural disasters when water-borne SSTIs become explosively frequent (e.g., hurricane Katrina in New Orleans in 2005, tsunami in Thailand in 2004, earthquake in Sichuan in 2008; [131]); (ii) or for digestive asymptomatic carriers and gastroenteritis, mostly associated with *A. caviae* and *A. veronii* [133], *A. caviae* being particularly associated with the environment of human beings since its density is greater in wastewater inflow than in outflow [134]; (iii) or for bacteremia, mainly caused by *A. caviae* and *A. veronii*, and indirectly associated with water through the gut from where these species originates most frequently.

Apart from water, aeromonads have been recovered from various types of ‘nurseries’ listed above, namely protozoa (amoeba), insects (mosquitoes) and worms (leeches), although the first two have not been studied in detail [135,136,137]. 

### 5.2. What Does *Aeromonas hydrophila* ATCC 7966^T^ Genome Teach Us?

The genome of virulent *A. hydrophila* ATCC 7966^T^ (NC_008570) reveals an exceptionally versatile organism with a large potential for pathogenic processes and persistence in aquatic environments. Its genome contains a single circular 4.7 Mb chromosome with 61.5% G+C content and encodes 5,195 predicted coding sequences, 10 ribosomal-gene operons and 128 tRNA that may be correlated with an ability to rapidly respond to changing environmental conditions [132]. Numerous extracellular components are encoded, proportional to its genome size, and designed to respond rapidly to environmental fluctuations (e.g., two-component signal proteins, methyl-accepting chemotaxis proteins), suggesting that, from a pathogenic point of view, the bacterium is critically concerned with sensing the external environment and that it may be involved in, sensing and responding to signals originating in the host environment. Its rRNA operon evolution includes at least in part a mosaic evolution by HGT of partial rRNA operon fragments [138,139]. The genome is characterized by comprehensive metabolic abilities. Besides complete primary metabolism pathways, secondary metabolism is very versatile including many chitinolytic activities, substantial formate catabolism, several enzymes for altering toxic compounds, phosphonates, xenobiotics, plastics, dyes or nitroaromatic compounds. The organism also seems capable of tolerance to heavy metals and stress response and detoxification functions with superoxide dismutases, catalase, arsenate reductase, *etc*. [132]. Transporter genes are numerous and compare with those of pseudomonads and vibrios. 

Few integrated regions have been observed, one of which includes lipopolysaccharide and carbohydrate synthesis that may be involved in chlorine resistance and the ability to form biofilm. Another one is a type 1 fimbriae gene cluster that may be involved in interaction with the host. Surprisingly, no transposase, resolvase or insertion sequence elements were found in *A. hydrophila* ATCC 7966^T^ genome, although these elements are usually associated with quick adaptation of free-living organisms.

The *A. hydrophila* ATCC 7966^T^ genome comprises a large panel of genes encoding virulence factors, including adhesion (type IV fimbriae and pili), toxins, iron acquisition, polar flagella, quorum-sensing regulation activators and antibiotic resistance among many others: all key processes for surviving in aquatic environments. Of note, genes encoding T3SS and one of its effectors (AexT) are absent although the role of the T3SS has been clearly established in the virulence of *A. hydrophila*. Since *A. hydrophila* ATCC 7966^T^ is one of the most virulent strains, it was suggested that the T3SS may be borne on a plasmid that may have been lost or that the absence of the T3SS is compensated by the presence of a type 6 secretion system or by the flagellar secretion apparatus, as described in *Y. enterocolitica* [132].

### 5.3. *Aeromonas salmonicida* subsp *salmonicida*: Evolution towards Specialization

**Figure 1:**
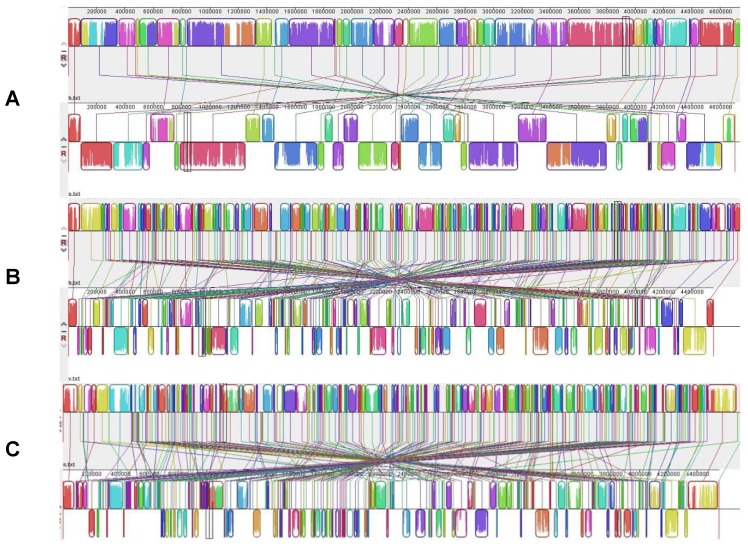
Gene alignment of genomes of *A. hydrophila*, *A. salmonicida* and *A. veronii* using MAUVE multiple alignments. Colored outlined blocks surround regions of the genome sequence that aligned to part of another genome. The colored bars inside the blocks are related to the level of sequence similarities. Lines link blocks with homology between two genomes. Genome pairs from top to bottom: (**A**) *A. hydrophila/A. salmonicida*; (**B**) *A. hydrophila/A. veronii*; (**C**) *A. salmonicida*/*A. veronii.*

*A. salmonicida* differs from other *Aeromonas* species by being psychrophilic, non-motile and having a pathogenic spectrum limited to fish. *A. salmonicida* appears to be an example of the evolution toward pathogen specificity for a particular host within a group of mainly opportunistic pathogens or commensal bacteria. The *A. salmonicida* A449 genome (NC_009348) consists of a single circular 4.7 Mb chromosome with a G+C% of 58.5 and 4388 genes encoding 4086 proteins, 9 rRNA operons, and 110 tRNA genes. There are additionally 2 large and 3 small plasmids. Compared to *A. hydrophila* ATCC 7966^T^, the *A. salmonicida* genome encodes a similar number of proteins although there has been gene gain or loss leading to a 9% difference in gene content [140,141]. This difference is characterized by a large number of mobile genetic elements (e.g., 88 IS) and of pseudogenes (170 compared to only 7 pseudogenes in *A. hydrophila* ATCC 7966^T^) in the *A. salmonicida* A449 genome and 5 plasmids, totalizing 0.34 Mb in genome size. A substantial number of IS acquisitions resulted in gene disruptions, some of them leading to the loss of cell surface structures such as flagella or pili, and of enzymatic activities [73,140]. Additionally, the *A. salmonicida* A449 genome differs by its global genome inversion around the origin of replication consecutive to large rearrangements and the acquisition of a large sequence absent from the *A. hydrophila* ATCC 7966^T^ genome, and probably acquired by transfer since the sequence is bounded by transposons (Figure 1A). Beside and inside the inverted region, the genomic synteny is, on the whole, respected (Figure 1A). 

Virulence factors include T3SS, a key factor for *A. salmonicida* virulence, and T6SS, although the latter seems to be not functional due to a key gene disruption. For both systems, genes are encoded either on the large plasmids or on the chromosome. The genome also contains genes encoding a secreted enzyme (e.g., collagenase), T2SS, iron acquisition, quorum sensing, (e.g., luxR) and pore-forming toxins (e.g., aerolysin). Interestingly, the A449 genome codes for an endotoxin 61% similar to the *Bacillus thuringiensis* insecticidal toxin CriET29, whose role remains unknown in *A. salmonicida*. Genes encoding adhesins like flagella, pili and surface layer are present, including the surface layer protein VapA, whose gene displays a low G+C% [141]. Attenuation of *A. salmonicida* by growth at high temperature is a result of IS insertion into the *vapA* gene [140]. Finally, genes of lateral and polar flagellae are present in the genome, but some of them are disrupted (e.g., lafA, flrA), which is consistent with the non-motile characteristic of the bacterium.

*A.* *salmonicida* evolved from a variable ancestral population to a rather genetically uniform host-adapted pathogen through accumulation of pseudogenes and IS, HGTs, plasmids and rearrangements [73].

### 5.4. Environment as a Training Ground: The Model of *A. veronii* with Leeches

The medicinal leech, *Hirudo verbana*, houses a remarkably simple two-member microbial community in the crop, (a pouch in its gut where ingested blood is stored): *A. veronii* and an uncultured *Rikenella*-like bacterium (*Bacteroidetes*). To date, only one other *Aeromonas* species, namely *Aeromonas jandaei*, has been recovered from the gut of a North American leech. The symbionts are thought to aid in digesting ingested blood, preventing other bacteria from colonizing the gut and providing essential nutrients. The 2 bacteria are organized in mixed-species polysaccharide embedded microcolonies that might form a biofilm associated with erythrocytes or that might float [135]. The complement system of the ingested blood remains active inside the crop and contributes to the specificity of the microbiota*, A. veronii* being a complement-resistant organism through its lipopolysaccharide layer. However, this factor is not enough to explain the symbiotic relationship because other complement resistant bacteria (e.g., *P. aeruginosa, Staphylococcus aureus*) have a markedly reduced ability to grow in the crop. Based on a signature-tagged mutagenesis, several genes have been involved in the interaction with the leech for a successful symbiotic relationship, including surface-expressed genes (e.g., lipopolysaccharide glycosylated surface proteins, exopolysaccharide formation, Braun’s major outer membrane protein), regulatory genes (e.g., regulating gene expression, enzymatic activity, protein synthesis rate), nutritional genes (e.g., threonine/serine transporter), host-interaction functions (e.g., T3S) or still unknown function genes [142,143]. In particular, the importance of regulatory genes for colonization shows the ability of *A. veronii* to recognize its host environment and regulate genes accordingly. Interestingly, the T3SS, also considered as a virulence factor [144,145], is essential for symbiosis with the leech and was detected in all *A. veronii* strains analyzed [146], except in the strain used for complete genome sequencing. It protects *A. veronii* against phagocytosis by leech hemocytes that circulate through the intraluminal fluid inside the crop, in order to maintain the symbiosis [147]. More generally, *A. veronii* seems to have an innate ability to colonize the gastrointestinal tract of blood feeding organisms (e.g., mosquitoes, vampire bats, leeches) and comprehensively to infect digestive tracts of multiple species with manifestations of interaction ranging from pathogenesis to mutualism [135,147].

Compared to the *A. hydrophila* ATCC 7966^T^ and *A. salmonicida* A449 genomes, *A. veronii* B565 (NC_015424) shows similar features with a single circular 4.55 Mb chromosome, a 58.7% G+C content, 4,057 protein coding genes, 10 rRNA operons and 102 tRNA genes [148]. Yet, several hundred genes present in *A. hydrophila* ATCC 7966^T^ and/or *A. salmonicida* A449 genomes were absent in *A. veronii* B565 genome (346, 329 and 366 genes, respectively), some of which are associated with mobile genetic elements. For example, 53% of the genes involved in interaction with the host were absent from *A. veronii* genome compared to *A. hydrophila* [142]. In addition, recombination events are frequent in *A. veronii* even in the T3SS genes. To date, no extensive genomic study, including comparative genomics, has been conducted on the *A. veronii* genome characteristics. We performed the alignment of *A. veronii* genome with those of *A. hydrophila* and *A. salmonicida* using MAUVE software (Figure 1B,C). In both comparisons, we observed extensive rearrangements with a loss of gene synteny. Considering the global genome conservation between *A. hydrophila* and *A. salmonicida*, this result suggested that *A. veronii* encountered a diversification process, which could be related to a variety of niches and hosts. The type of genes involved in *A. veronii* mutualistic strains is similar to those found in pathogenic strains [144,147]. Although it remains unclear why the same function acts as colonizing in invertebrates and pathogenic in human or fish, the leech colonization may be a good ‘training course’ for *A. veronii* in pre-adaptation to digestive tract colonization, resistance to complement and serum lysis and resistance to phagocytosis. These qualities may in turn be used to sustain human infection, especially for gastroenteritis and bacteremia.

## 6. Is *Brucella* an *Ochrobactrum* with Reduced Genome?

The order *Rhizobiales* within *Alphaproteobacteria* is of interest to study the emergence of pathogens and the OBP behavior due both to the range of ecological niches they inhabit, and to the range of their interactions with eukaryotic hosts. While many of these interactions are pathogenic in nature, including a variety of diseases in humans and other animals, there are also many interactions of a symbiotic and beneficial nature. *Brucella* and *Bartonella* are the causative agents of human diseases while the genera *Agrobacterium* and *Sinorhizobium* or *Rhizobium* include pathogens and symbionts of plants. These ‘specialized’ genera are extensively studied at the genomic, genetic and physiological levels. Here, we focus on the versatile genus *Ochrobactrum* as the ideal model to study the adaptation to different ecological niches and emergence of specialized lifestyles, such as the strict pathogenicity of *Brucella*. The rule that free-living generalist bacteria have larger genomes than closely related bacteria with specialist behavior is now clearly established. Comparative genomics between the strictly intracellular pathogens of the genus *Brucella* and the mild OBPs of the genus *Ochrobactrum* illustrate this general rule.

The genus *Ochrobactrum* comprises highly versatile bacteria with the ability to colonize a wide variety of habitats, from hostile environments such as polluted soil, to plants, nematodes, insects, animals and man. Some species have been isolated from leguminosae nodules. An increasing number of studies report the isolation of *O. anthropi* and *O. intermedium* from clinical specimens, especially from immunocompromised patients or during HAIs related to indwelling devices, dialysis or surgery [24]. Even if its genome contains a complete homolog of a well-known virulence operon (*virB*) on a large transferable plasmid, *O. anthropi* is described as a mild human OBP, worrisome mainly for its exceptional resistance to antimicrobial agents. However, the *virB* operon is the major determinant of virulence for strict pathogens related to the genus *Ochrobactrum*. It is the main support for DNA transfer and for phytopathogenicity in *Agrobacterium tumefaciens*. In *Brucella* spp., it allows intra-macrophagic survival and multiplication of the bacterium in mammals [149]. The case of *Ochrobactrum* shows that acquisition of a major factor of virulence is not enough to become a strict pathogen.

### 6.1. Genomics of Brucellaceae

The complete genome of *O. anthropi* ATCC 49188^T^ has been recently but briefly reported [150]. Similar to other Rhizobiales [151] and to other members of the genus *Ochrobactrum* [57], the genome of *O. anthropi* ATCC 49188^T^ consists of multiple circular chromosomes, a replicon of about 2.9 Mb with the prototypical bacterial chromosome origin of replication (*oriC*), and a second replicon of about 1.9 Mb with a repABC origin commonly found in other secondary chromosomes and plasmids of the Rhizobiales [150]. In addition, the *O. anthropi* ATCC 49188^T^ genome contains four plasmids carrying genes related to stabilization factors, including genes encoding ParB-like, PilT-type and toxin-antitoxin addiction systems. Plasmids include a large number of genes encoding transposases, integrases and several transporters, suggesting that they contribute to bacterial fitness via genes acquired by HGT [150]. Genome size appears to be exceptionally variable (from 5.06 to 8.12 Mb) in the species *O. anthropi*, variations bearing mostly on number and size of plasmids [57]. The strain-specific plasmid content stresses the role of plasmids for adaptation of each strain to a particular niche, therefore supporting versatility in the species *O*. *anthropi*.

**Figure 2:**
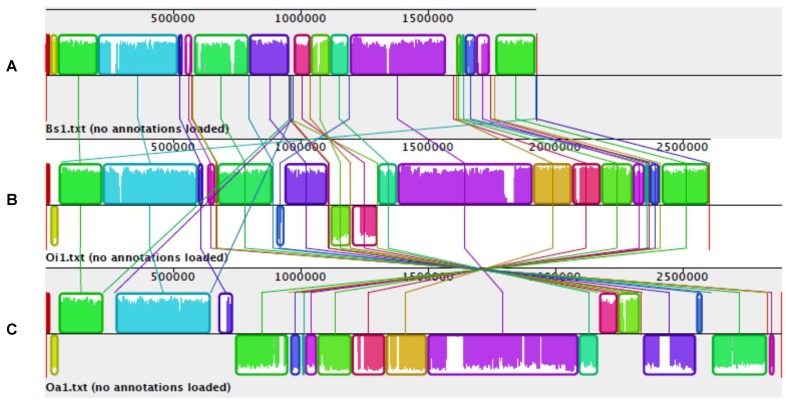
Gene alignment of large chromosome (ChrI) of *B. suis*, *O. intermedium* and *O. anthropi* using MAUVE multiple alignments. Colored outlined blocks surround regions of the genome sequence that aligned to part of another genome. The colored bars inside the blocks are related to the level of sequence similarities. Lines link blocks with homology between two genomes. Genome from top to bottom: (**A**) *B. suis*; (**B**) *O. intermedium*; and (**C**) *O. anthropi*.

**Figure 3:**
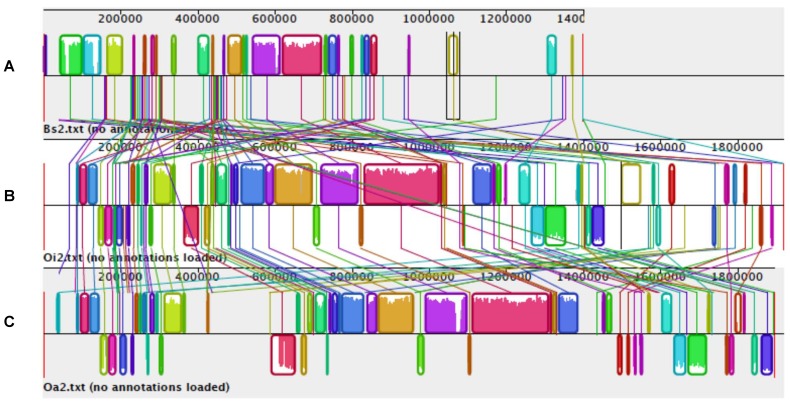
Gene alignment of small chromosome (ChrII) of *B. suis*, *O. intermedium* and *O. anthropi* using MAUVE multiple alignments. Colored outlined blocks surround regions of the genome sequence that aligned to part of another genome. The colored bars inside the blocks are related to the level of sequence similarities. Lines link blocks with homology between two genomes. Genome from top to bottom: (**A**) *B. suis*; (**B**) *O. intermedium*; and (**C**) *O. anthropi*.

The draft sequence of *O. intermedium* is now available, allowing a preliminary comparison of *Brucella suis*, *O. anthropi* and *O. intermedium* genomes. The alignment performed with the MAUVE software is shown in Figure 2 and Figure 3. The large chromosomes (chr I) of the 3 species are similar and roughly colinear but display clear differences in size related to indels scattered in the sequences (Figure 2). Compared to *O. anthropi*, chr I of *O. intermedium* is very similar in content but is smaller by about 300 kb, suggesting a reductive process confirmed by a higher % of pseudogenes than observed in *O. anthropi*. Moreover, *O. intermedium* compared to *O. anthropi* presents a large inversion involving two thirds of the genome. Surprisingly, colinearity is respected between *O. intermedium* and *B. suis* genomes, the main differences between them corresponding to deletions leading to a genome size smaller by about 700 kb for *B. suis* (Figure 2). This confirms the previously described genome degradation in brucellae [152]. The major deletion observed by MAUVE comparison corresponds to a large fragment of about 430 kb, present in the *Ochrobactrum* but not the *Brucella* chr I. About half of the corresponding genes are absent from *Brucella* spp. genomes and another half are inserted in chr II of *B. suis*. Surprisingly, some of the latter genes are inserted in chr II of *B. suis* ATCC 23445 and in chr I of *B. suis* 1330. This result confirms that recombination events occurred between the two chromosomes in brucellae genomes as previously described for recombination between *rrn* operons [78]. Comparison of the small chromosomes (chr II) of *B. suis*, *O. anthropi* and *O. intermedium* genomes (Figure 3) shows more indel events and lack of synteny than observed for chr I. Again. the chr II of *B. suis* is smaller than those of *O. anthropi* and *O. intermedium*. Despite indels and rearrangements, chr II of *O. anthropi* and *O. intermedium* remain globally colinear while *B. suis* displays an extensively modified chr II. Clearly, chr II supports the genomic diversity in *Brucellaceae* and probably ensures the adaptation to diverse niches. 

Plasmid POAN01 of *O. anthropi* includes a complete set of type IV secretion system genes (operon *virB*) whereas this operon is found on the second chromosome of *O. intermedium*. This suggests the transfer of virulence traits from a disposable to an essential replicon leading to immortalization of the function. 

Although previous studies have supported the notion of *Brucella* as a monospecies genus, it is now accepted that the genus *Brucella* is divided into ten species, named according to their preferential hosts, except for *Brucella inopinata*, for which a natural host remains unknown. Genome sequencing of *Brucella melitensis*, and *B. suis*, the most pathogenic among brucellae, demonstrated a high level of similarity between the two genomes, with more than 90% of genes having more than 98% nucleotide identity [153]. It has been proposed that the unique complement of pseudogenes in each of the *Brucella* species may contribute to their differing degrees of infectivity and host preference [154]. This suggested that the differential genome degradation among brucellae confers their specific pathogenic behavior. Compared to *B. suis* and *B. melitensis*, the biology of *Brucella ovis*, which is non-pathogenic for humans, but closely associated to and pathogenic for sheep, appears to be in part the result of genome degradation [152], as suggested by the % of pseudogenes (Table 3). This genome reduction correlates with the adaptation to a narrower niche in a unique host. Moreover, the majority of human brucellosis cases occur via ingestion of contaminated dairy products, except for *B. ovis*. This suggests that this species has lost the ability to infect via the oral route in relation with genome degradation, particularly the loss of urease that is required for survival of stomach acidity by *Brucella* spp. [155]. By contrast, the genome of the OBP *O. anthropi* genome displays a very low % of pseudogenes, evoking the conservation of a large repertoire adapted to life in versatile niches.

### 6.2. Real-Time Genomic Reduction in an *Ochrobacterium intermedium* Clone

In a previous study, we analyzed clonal strains chronologically isolated from respiratory tract samples of a patient with chronic *O. intermedium* carriage over a one-year period. We observed the loss of one *rrn* copy and a large genomic deletion that occurred between the second (AV2) and the third isolate (ADV3) obtained from the patient over a period of one month. *In vitro*, variations in colony aspect and growth rate were observed between ADV2 and ADV3. The new genomic organization and phenotype were maintained in four subsequent isolates obtained over a 4-month period. This suggests that the new genomic organization gave selective advantage to the strain and that bacterial life in a very narrow ecological niche, in this case the human respiratory tract, led to a reductive evolution process [90]. The rearrangement consisted in a 150-kb deletion corresponding to the whole genomic region comprised between the two *rrn* copies of the small chromosome. The deletion occurred by homologous recombination between the 2 *rrn* copies and resulted in the loss of one copy. Interestingly, the genomic skeleton obtained after the deletion event occurred evoked the genomic structure of *Brucella* spp. with reduced genomic size and only 3 *rrn* operons. 

This report of genomic reduction in real time, taken together with the preliminary results concerning the total sequence of *O. intermedium i.e.*, reduction of chr I, % of pseudogenes and ‘immortalization’ of *virB* on chr I, suggest that this bacterium might be involved in a similar specialization process as that encountered by *Brucella* in its own evolution. Data at the population level confirmed that most strains of *O. intermedium* are isolated from human beings (Aujoulat, personal communication).

## 7. Conclusive Remarks

In this overview of OBPs, we detailed the genomics of three groups of organisms: several strains of *P. aeruginosa*, 3 species of aeromonads and 2 species of *Ochrobactrum*, compared to the related strict pathogen, *Brucella* spp. All have, as is probably the case in most OBPs, a common genome content reflecting the versatility of life-styles. However, to achieve this versatility, each OBP has its own genome organization and dynamics.

**Table 3:** Features of selected *Brucellaceae* genomes obtained from NCBI and PATRIC database.

**Species** Strain RefSeq or INSDC	***B. ovis*** ATCC 25840^T^ NC_009505–009504		***B. suis*** 1330 CP002997–002998		***B. melitensis*** **16M** NZ_ACJL		***O. anthropi*** ATCC 49188^T^ NC_009667–09668		O. intermedium * LMG 3301^T^ NZ_ACQA	
	***Chr I***	***Chr II***	***Chr I***	***Chr II***	***Chr I***	***Chr II***	***Chr I***	***Chr II***	***Chr I***	***Chr II***
Size (bp)	2,111,370	1,164,20	2,107,792	1,207,381	2,117,144	1,177,787	2,887,297	1,895,911	NA	NA
GC content (%)	57.2	57.2	57.2	57.3	56	56	57.2	57.3	57	57
Protein coding	2,890		3,272		3,165		4,424		4,363	
rRNA operons	2	1	2	1	2	2	2	1	2	2
N° of tRNAs	53		55		54		73		NA	
N° pseudogenes	244		62		172		31		77	
% pseudogenes	7.8		1.8		5.1		0.7		1.7	

* draft sequence; NA, not available; *chr I* for chromosome 1, *chr II* for chromosome 2.

In the species *P. aeruginosa*, the core/accessory genome mosaicism and the notable presence of non-essential genes in the core genome (such as genes encoding virulence factors) account for the generalist behavior of this virulent opportunistic pathogen. Its adaptation to particular niches could be related either to acquisition of specific genes or to expression of phenotypic traits. Consequently, despite its versatility, the species *P. aeruginosa* appears to be quite homogeneous. The whole species appears both generalist and specialized with no sign that a highly specialized clone is emerging. The only exception appears to be the hypervirulent strain LESB58 that displays a higher number of pseudogenes than other strains, suggesting that LESB58 is undergoing a genomic reduction due to its specialization. 

Versatility is also observed in aeromonads that display a wide array of metabolic capabilities, genes involved in general interactions that may also be virulence factors, ‘environment sensors’ and the outfit allowing a rapid response to environmental fluctuations. This was observed in the whole genus *Aeromonas*, comprising related species. For instance, *A. hydrophila* and *A. salmonicida* have closely related genomes that share more than 90% of their content. *A. salmonicida* is a specialized pathogen of fish, and compared to the OBP *A. hydrophila,* its genome displays signs of specialization to a narrow niche, e.g., a high number of pseudogenes and IS that can be considered as a prelude to genome reduction. The genus *Aeromonas* can be considered as a tight complex of species among which one has emerged through adaptive specialization to a niche. The phylogenetic relatedness between *Ochrobactrum* and the strict pathogen *Brucella* provides another example of accomplished genomic reduction related to the emergence of specialized species associated to a narrow niche from a complex of versatile OBP species.

Besides accumulation of pseudogenes preceding genome reduction and outbreak of IS foretelling niche specialization, the genome evolution of *P. aeruginosa*, aeromonads and *Ochrobactrum* spp. is mainly due to the acquisition of mobile genetic elements, gene gain and loss, recombination events, and large rearrangements. 

We believe the modes of genomic evolution illustrated by these three detailed models are common to most OBPs, even if sufficient genomic data is still lacking to confirm this hypothesis. Nevertheless, to understand the dynamics of pathogens' evolution, genomic data should be considered in the light of the population structure. Populations of versatile opportunistic pathogens are structured in clonal complexes rather than in delineated ‘true’ species [54]. However, some populations of opportunistic pathogens display emerging clones associated to human beings, as described for *O. anthropi* and *A. tumefaciens* among others [18,24]. Adaptation to humans coincides with a functional specialization revealed by modifications in both genomic and population structures. Besides the acquisition of specialized virulence factors, this adaptation-driven speciation could be a major mechanism that prepares for the emergence of true pathogens.
